# A Legendre Neural Network-Based Approach to Multiparameter Identification of Traffic Loads Across the Full Spatiotemporal Domain

**DOI:** 10.3390/s24237785

**Published:** 2024-12-05

**Authors:** He Zhang, Ruihong Shen, Yuhui Zhou, Cun Zhang, Zhicheng Zhang

**Affiliations:** 1 College of Civil Engineering and Architecture, Zhejiang University, Hangzhou 310058, China; zjuzhanghe@zju.edu.cn (H.Z.); shenrh@zju.edu.cn (R.S.); jszzc@zju.edu.cn (Z.Z.); 2 Center for Balance Architecture, Zhejiang University, Hangzhou 310058, China; 3 China United Engineering Corporation Limited, Hangzhou 310052, China; 4 College of Hydraulic and Civil Engineering, Tibet Agriculture and Animal Husbandry University, Linzhi 860000, China; zhangcun@xza.edu.cn; 5 Research Center of Civil, Hydraulic and Power Engineering of Tibet, Linzhi 860000, China

**Keywords:** traffic load identification, Legendre neural network, network optimization, artificial intelligence

## Abstract

The accurate identification of traffic loads acting on bridges provides an effective basis for the traffic control and operation of in-service bridges. To improve the efficiency and accuracy of loading identification, we propose an efficient multiparameter identification method with a Legendre neural network (LNN) for the monitoring of traffic loads across the full spatiotemporal domain. Compared to conventional studies that suffer from ill-posed problems and neural network-based means that lack a physically interpretable model, with the proposed strategy, both the explicit expression and time histories of the traffic load can be simultaneously obtained. Meanwhile, inaccurate load identification at the bridge’s supports, which is caused by ill-posed problems, does not exist in the identification process using the LNN. After the training and optimization of the LNN, its identification accuracy for speed and the magnitude of forces reached 98.6% and 98.3%, respectively. The results suggest that an identification method with a well-trained LNN is insensitive to noise.

## 1. Introduction

Traffic loads are the primary dynamic exciting source for bridges during their operations, and their long-term actions can cause vibrations and induce fatigue failures [1,2,3,4]. With the development of the economy and the increase in traffic flow on bridges, traffic overloading has become a major potential safety hazard for bridges and has even led to the collapse of bridges in some extreme cases [5,6,7,8]. Therefore, obtaining traffic loading information is crucial for the traffic control and safety insurance of bridges.

Traffic load identification methods primarily include two categories: direct detection methods and indirect detection methods. Direct detection methods involve using weight-in-motion (WIM) systems to monitor vehicle gross weight and axle weight, as well as other parameters [9,10,11]. These systems enable non-intrusive weight measurement without stopping vehicles, ensuring efficient operation with minimal disruption to traffic flow [12]. Another key advantage of WIM systems is their ability to be deployed ahead of bridges, enabling proactive risk management. In cases where the risk of overloading is predicted to be high, vehicles can be redirected to alternative routes before reaching the bridge, thus mitigating the risk of overload [13,14]. Currently, commonly used WIM systems include bending plates, load cells, and piezoelectric systems. However, the initial installation of WIM systems and their equipment are costly, and maintenance expenses are also relatively high [15,16].

Indirect detection methods rely on the measured vibration responses of the bridge under the excitation of traffic loads and can identify the traffic load through the inverse solution of the motion differential equations of the vehicle–bridge system [17]. Some of these methods are derived from analytical models, where the equations of motion are inversely solved to determine an analytical solution for the traffic load. Law et al. [18] introduced the time domain method (TDM), in which moving forces are represented as a small time-step function. The motion equations are then solved in the time domain to obtain the time histories of the forces. Later, Law [19] proposed a frequency–time domain method, where the equations of motion expressed in modal coordinates are subjected to Fourier transform to establish a relationship between responses and forces in the frequency domain. However, these methods exhibit sensitivity to noise and significant fluctuations in accuracy due to the ill-posed nature of the inverse problem. To address this issue, some researchers have applied the Tikhonov regularization method to the TDM [20], enhancing the accuracy of load identification in noisy environments. Zhang [21] further employed the L-curve method to select an optimal regularization parameter while solving the bridge’s motion equation inversely with the Tikhonov regularization, thereby making the identification process less susceptible to noise interference. Xiao et al. [22] improved the stiffness separation method to reduce the computational effort for solving large-scale matrix inversion problems.

Due to the complexity of vehicle–bridge interaction models, solving their motion equations analytically is challenging. Therefore, various identification methods have been developed based on finite element methods. Compared to identification strategies based on analytical models, the errors caused by modal truncation in the dynamic response and modal coordinate transformation are avoided with finite element methods [23,24]. Law et al. [25] introduced the optimal state estimation method, with a bridge modeled in the state space, and conducted moving load identification using the dynamic programming optimization technique. Pinkaew et al. [26,27] developed the updated static component technique, which separates the load into static and dynamic components and corrects the moving force by updating the static component.

However, such conventional load identification methods still have ill-posed problems while solving motion equations inversely with multiple difficult-to-identify parameters [28,29,30,31]. Researchers have introduced machine learning methods for traffic load identification with the advantage of strong robustness and nonlinear mapping abilities [32,33,34,35]. Yang et al. [23] investigated the feasibility of applying Back Propagation (BP) neural networks to solve the moving force identification problem under different combinations of activation functions and algorithms, and the results showed that the accuracy of the method is satisfactory. Zhou et al. [36] applied deep convolutional neural networks to identify the acceleration response from noise-contaminated moving loads and obtained results with high accuracy. Zhou et al. [37] proposed a Recurrent Neural Network (RNN) model that can directly use the original dynamic response to identify unknown impact load histories. The temporal relationships between impact loading and the dynamic responses of structures are established by the RNN, which may reflect the time-varying features of the impact load. However, only the time histories of traffic are obtained with most machine methods from the measured dynamic responses of the bridge. The relationship between the moving force and the strain of bridges established by machine learning methods is hidden in the model and cannot be observed or understood by researchers. Moreover, each time machine learning methods are applied to a new bridge for the identification of the traffic load, it is necessary to establish a new database and train the models again, which is time consuming.

A novel method with a Legendre neural network (LNN) was developed for traffic load identification [38,39]. The nonlinear equations of motion are transformed into linear equations through the LNN. Then, the LNN is trained to obtain the approximate expressions of magnitude and speed of force. Hence, not only the time history of the traffic load but also the explicit expression of the traffic load will be obtained. This makes it possible to understand the effect of strain response on loading, which improves the interpretability of the model in explaining the complex vibration processes. Meanwhile, the explicit expression obtained by the LNN is applicable to different bridges of the same type. When applying this approach to a new bridge with the same physical parameters but a different length, it does not need to retrain the LNN with a new database like most other neural networks, which greatly improves the applicability and efficiency of the method.

The remainder of the paper is organized as follows: Section 2 introduces the proposed LNN for moving load identification. Section 3 presents the hyperparameter optimizations and training of the LNN. In Section 4, the proposed methodology is validated both numerically and experimentally for a three-span continuous beam. Section 5 discusses the influences of some factors on the accuracy of loading identification, and in Section 6, some conclusions are derived.

## 2. Methodology

**Figure 1:**
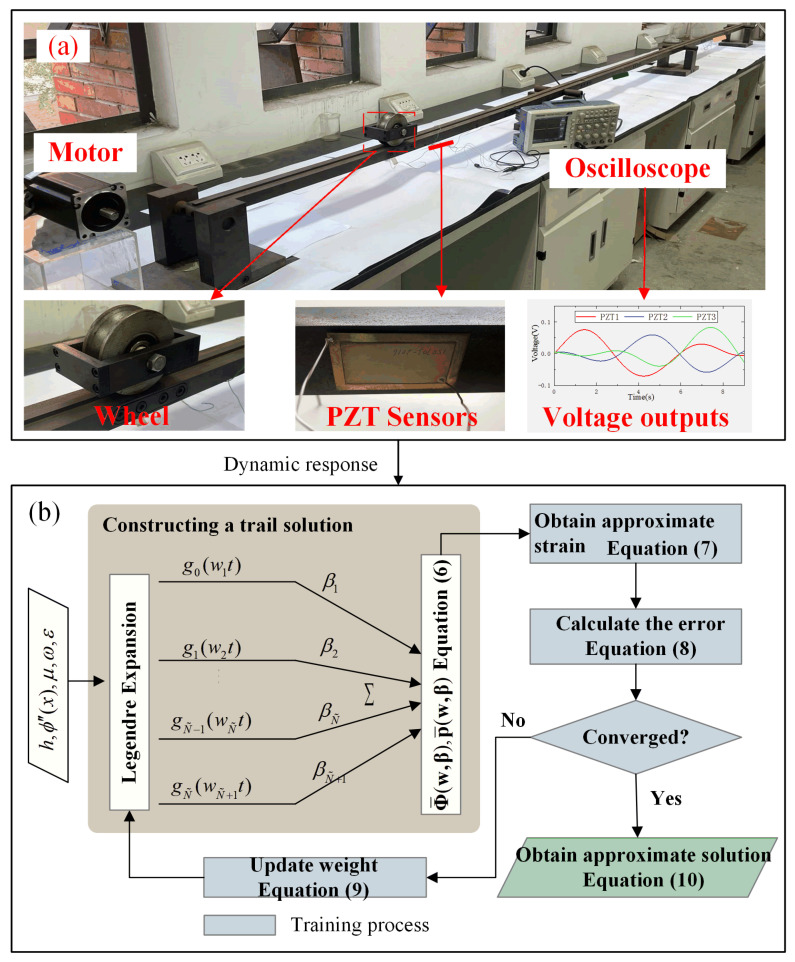
Identification of moving force using Legendre neural network: (**a**) picture of the experiments; (**b**) flowchart of moving force identification with the LNN.

In the identification of traffic loading, the dynamic strains of the beam are obtained from the voltage outputs of a series of lead zirconium titanate (PZT) sensor arrays. With these dynamic strains, the approximate expression of the magnitude and speed of the moving load will be obtained with the LNN. The PZT sensors are arranged along the underside of the beam to measure the time history strains (Figure 1a). According to the electromechanical analyses [40,41,42,43], the time history of the strains is obtained as follows:

(1) $$\varepsilon (x,t)=-\frac{h}{a}\left(\frac{{\bar{\mu }}_{33}s_{11}}{h_{p}d_{31}}V(t)+\frac{s_{11}}{d_{31}A_{\mathrm{PZT}}R}\int_{0}^{t}V(\tau )d\tau \right)$$

where *h* and *a* represent the distance from the composite section and PZT sensor center to the underside of the beam, respectively; ${\bar{\mu }}_{33}$ represents the effective dielectric permittivity; $s_{11}$, $d_{31}$, and $h_{p}$ denote the elastic compliance coefficient, the piezoelectric constant, and the thickness of the PZT sensor; *R* denotes the resistance; $A_{\mathrm{PZT}}$ denotes the PZT sensor area; and *V*(*t*) denotes the voltage of PZT sensor.

According to the Euler–Bernoulli beam theory, the relationships between the strains and traffic loading are obtained according to the equation of motion:

(2) $$\rho A\frac{\partial^{2}w(x,t)}{\partial t^{2}}+EI\frac{\partial^{4}w(x,t)}{\partial x^{4}}=\delta (x-vt)p(t)$$

where *ρ*, *A*, *EI*, and w(x,t) denote the line mass density, the cross-sectional area, the bending stiffness, and the displacement of the continuous beam, respectively; $x_{t}$ is the location of the force at time *t*; *v* and *p*(*t*) denote the speed and magnitude of the force; and δ(t) is the Dirac delta function.

Using the modal superposition method, displacement can be expressed as $w(x,t)=\sum_{n=1}^{\infty }\phi_{n}(x)\eta_{n}(t)$. Substituting this expression of displacement into Equation (2), and multiplying by $\phi_{n}(x)$, integrating with respect to x over the interval from 0 to *L*, and applying the orthogonality conditions, we will obtain

(3) $${\ddot{\eta }}_{n}(t)+\omega_{n}^{2}\eta_{n}(t)=\frac{1}{\mu_{n}}p(t)\phi_{n}(vt)$$

where $\omega_{n}$ and $\mu_{n}=\int_{0}^{l}\phi_{n}^{2}\rho Adx$ represent the *n*th modal frequency and the modal mass, respectively.

The solution for $\eta_{n}(t)$ can be obtained as follows:

(4) $$\eta_{n}(t)=\frac{1}{\mu_{n}\omega_{n}}\int_{0}^{t}p(t)\phi_{n}(vt)\sin[\omega_{n}(t-\tau )]d\tau$$

Substituting Equation (4) into the expression of displacement, the displacement could be obtained. For the small deformation beam, the strain can be calculated as follows:

(5) $$\varepsilon (x,t)=-h\frac{\partial^{2}w(x,t)}{\partial x^{2}}=-\sum_{n=1}^{\infty }h\phi_{n}^{\prime \prime }(x)\frac{p(t)}{\mu_{n}\omega_{n}}\int_{0}^{t}\phi_{n}(vt)\sin\left[\omega_{n}(t-\tau )\right]d\tau$$

where ε denotes the strains measured by the sensors or calculated by simulation.

Equation (5) represents the strain with the speed v(t) and magnitude p(t) of the moving force to be identified. The speed information is included in the mode shape function, which has a complex expression for continuous beams. Therefore, it is difficult to obtain explicit expressions of the time-varying magnitude and speed of the moving load simultaneously. In view of this, an LNN is applied to obtain the explicit expression of the traffic load [38,39].

For the LNN, trial expressions of $\phi_{n}(x_{p}(t))$ and p(t) are assumed to be a linear weighted combination of Legendre polynomials (Figure 1b):

(6a) $$\bar{\Phi }(x_{p}(t))=\left[\begin{array}{c} {\bar{\phi }}_{1}(x_{p}(t))\\ {\bar{\phi }}_{2}(x_{p}(t))\\ \ldots \\ {\bar{\phi }}_{n}(x_{p}(t)) \end{array}\right]=\left[\begin{array}{c} \sum_{n=0}^{N}g_{n}(W_{n}t)\beta_{1n}\\ \sum_{n=0}^{N}g_{n}(W_{n}t)\beta_{2n}\\ \ldots \\ \sum_{n=0}^{N}g_{n}(W_{n}t)\beta_{Nn} \end{array}\right]$$

(6b) $$\bar{p}(t)=\sum_{n=0}^{N}g_{n}(W_{n}t)\beta_{(N+1)n}$$

(6c) $$g_{1}(t)=1;\;g_{n+1}(t)=\frac{1}{2^{n}n!}\frac{d^{n}}{dt^{n}}\left[{\left(t^{2}-1\right)}^{n}\right]$$

where W denotes the weight of the input layer, β denotes the weight of the hidden layer, and $g_{n}$ denotes the Legendre orthogonal polynomial of the *n*th mode in the real interval [−1, 1].

Then, the approximate strain of the bridge will be obtained with the trial expressions of $\phi_{n}(x_{p}(t))$ and p(t). The structural parameters $h,\;\phi^{\prime \prime }(x),\;\mu ,\;\omega$ and the dynamic strain ε of the structure are fed into the LNN, while the traffic loadings $p,\phi_{n}(x_{p}(t))$ are the target output; therefore, the strain will be

(7) $$\bar{\varepsilon }(x_{l},t_{j})=-\sum_{n=1}^{\infty }\frac{h\phi^{\prime \prime }(x)}{\mu_{n}\omega_{n}}\int_{0}^{t}\bar{p}(\tau ){\bar{\phi }}_{n}(x_{p}(\tau ))\sin\left[\omega_{n}(t-\tau )\right]d\tau$$

The mean squared error between the approximate strain $\bar{\varepsilon }(x_{l},t_{j})$ and the actual strain ε is defined as

(8) $$MSE=\frac{1}{M}\sum_{j=0}^{M}\sum_{i=0}^{3}{\left({\bar{\varepsilon }}_{i}(x_{l},t_{j})-\varepsilon_{i}\right)}^{2}$$

The weights of the Legendre polynomials are updated to minimize the error of Equation (8). In the iteration process, the weights of the (*k* + 1)th step are modified as

(9) $$\begin{array}{l} W_{n}^{k+1}=W_{n}^{k}+\Delta W_{n}^{k}=W_{n}^{k}-\eta_{l}\frac{\partial E}{\partial W_{n}^{k}}\\ \beta_{n}^{k+1}=\beta_{n}^{k}+\Delta \beta_{n}^{k}=\beta_{n}^{k}-\eta_{l}\frac{\partial E}{\partial \beta_{n}^{k}} \end{array}$$

where $\eta_{l}$ is the learning rate.

The weights of neurons in the LNN will be updated until the set precision MSE≤0.01 is reached. The LNN training is considered completed with the final weight series of $\left(w_{n}^{\bar{k}},\beta_{n}^{\bar{k}}\right)$. The solutions of p(t) and $\phi_{n}(x_{p}(t))$ will be

(10) $$\begin{array}{l} \bar{\Phi }(x_{p}(t))=\left[\begin{array}{c} {\bar{\phi }}_{1}(x_{p}(t))\\ {\bar{\phi }}_{2}(x_{p}(t))\\ \ldots \\ {\bar{\phi }}_{n}(x_{p}(t)) \end{array}\right]=\left[\begin{array}{c} \sum_{n=0}^{N}g_{n}(W_{n}^{\bar{k}}t)\beta_{1n}^{\bar{k}}\\ \sum_{n=0}^{N}g_{n}(W_{n}^{\bar{k}}t)\beta_{2n}^{\bar{k}}\\ \ldots \\ \sum_{n=0}^{N}g_{n}(W_{n}^{\bar{k}}t)\beta_{Nn}^{\bar{k}} \end{array}\right]\\ \bar{p}(t)=\sum_{n=0}^{N}g_{n}(W_{n}^{\bar{k}}t)\beta_{(N+1)n}^{\bar{k}} \end{array}$$

Then, the location and speed of the moving force at time *t* will be

(11) $$x_{p}(t)={\phi_{n}}^{-1}({\bar{\phi }}_{n}(x_{p}(t)));\;v(t)=x_{p}^{\prime }(t)$$

## 3. Training of the Legendre Neural Networks and Hyperparameter Optimizations

To optimize the proposed neural network and validate its effectiveness for traffic load identification, the training processes of the LNN are conducted using a three-span continuous beam. The beam consists of three spans, each spanning 1.5 m, and its other parameters are detailed in Table 1. To realize the LNN training process, we establish a database comprising 824 groups with a total of 74,160 samples, which is divided into training, validation, and test datasets. Among them, the training dataset contains 45,000 samples, the validation dataset contains 14,580 samples, and the test dataset contains 14,580 samples. Additionally, during LNN training, the patience is set to 5 epochs, meaning that if the performance on the validation set does not improve over 5 consecutive epochs, the training will be stopped. The learning rate is set to 0.01. The values of other force parameters are detailed in Table 2.

When the database is established, the relationship between the strains of the beam and the forces will be established with the data collected. To refine the neural network for adaptation to our task, the optimal hyperparameters, including the learning rate and sample quantity, are tuned during the training process of the LNN.

**Figure 2:**
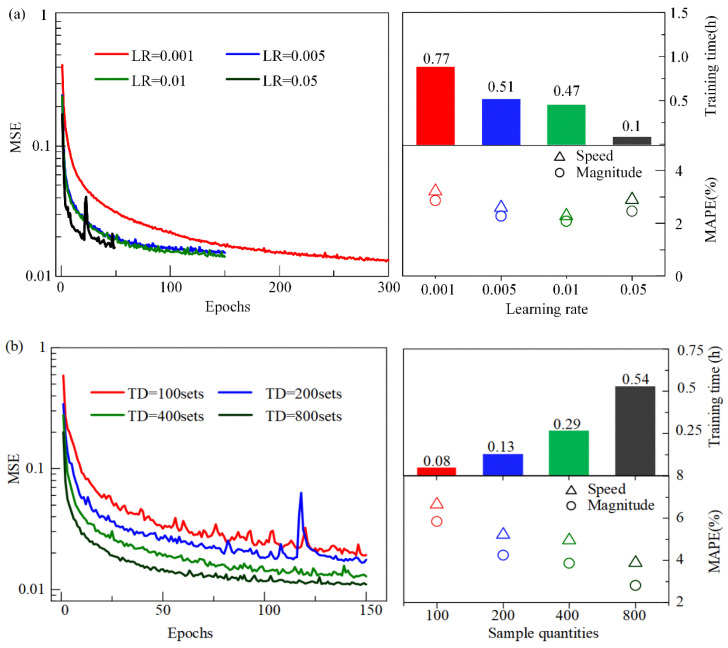
The convergence process and the training time of the LNN: (**a**) four cases with different learning rates; (**b**) four cases with different sample quantities.

The learning rate is responsible for controlling the convergence rate of the LNN during training. A higher learning rate causes the weights of the LNN to update more quickly. An appropriate learning rate can aid in speeding up the network’s convergence and improving the precision of load identification with the LNN. However, if the value of the learning rate is set too low, the LNN will converge extremely slowly, and the training process will be time consuming. The network may also fall into local minima, leading to low identification accuracy of the traffic load. Conversely, when the value of the learning rate is set too large, each adjustment to the weights becomes too significant, resulting in the loss function values oscillating around the minimum or failing to converge. Here, the mean squared error (MSE) of the training set, the training time, and the Mean Absolute Percentage Errors (MAPEs) of the validation set are utilized to evaluate the convergence processes, training speed, and identification accuracy of the four LNNs (Figure 2a). The results show that the higher the learning rate, the faster the network’s convergence speed and the lower the training time cost. In addition, the MAPEs of the validation set indicate that when the learning rate increases from 0.001 to 0.01, the accuracy of the identified traffic load with the LNN becomes better, but it becomes worse when the value further increases from 0.01 to 0.05. In summary, to ensure the best efficiency of the LNN training process and moving load identification accuracy, the optimal learning rate is herein assumed to be 0.01.

On the other hand, the identification accuracy for moving loads is also strongly related to the training sample quantity. Generally, increasing the number of training samples can improve the accuracy of the model, as more samples provide richer data features, helping the network learn the underlying patterns in the data more accurately. However, it will also increase the training time, as the network needs to process more data. In practical applications, it is expensive and time-consuming to establish a large number of samples through in situ tests, experiments, or theoretical simulations, and thus the sample quantities for network training may be relatively limited. Herein, four databases are established with different numbers of training samples to evaluate the effect of sample quantity on the performance of the LNNs. It is observed that the training time of the network increases significantly with more training samples (Figure 2b). When the networks are well trained, the identified results of the test set show that the identified moving loads with the LNN with more training samples are closer to the applied ones (Figure 2b). In addition, the precision and stability of the identified moving force with the LNN improve when the training samples increase from 100 to 800 sets. When the training sample quantities are greater than 200, the MAPEs of the identified moving force with the LNN are less than 5%. In conclusion, the optimal training sample quantity is 800 groups to ensure the best efficiency of the LNN training process and moving load identification accuracy.

## 4. Verification of the Traffic Load Identification Method

To validate the precision of the identification method with the LNN, numerical simulations and experiments are conducted with the optimized LNN.

### 4.1. Numerical Verification

**Figure 3:**
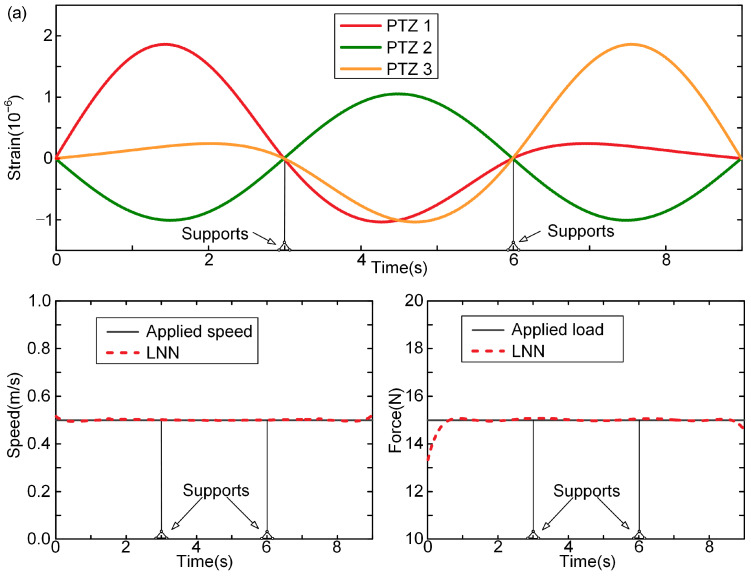

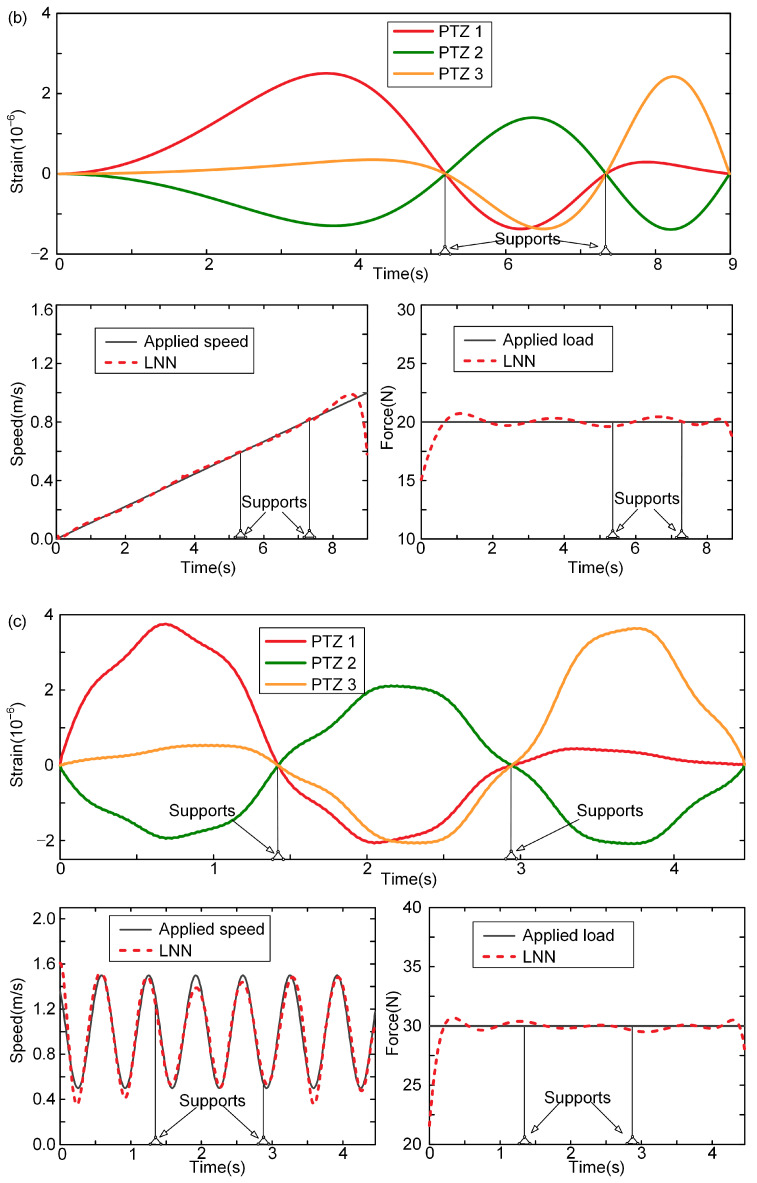
Identified speed and force of loading with LNN in three cases: (**a**) case 1, constant speed; (**b**) case 2, speed is uniform acceleration; (**c**) case 3, sinusoidal variation in speed.

With the measured dynamic strain information of the bridge fed into the LNN, the moving force can be identified with the LNN, in which the precisions of the identified loadings are verified through three cases. In these cases, the speeds of the force are assumed to be constant, uniformly accelerated, and harmonically varying. Figure 3 shows the time histories of the beam strain response for the three cases and the comparison of the loads identified by the LNN with the applied loads. In the first loading case, the applied loading is assumed to move at a speed of 0.5 m/s and remain uniform in the time domain (Figure 3a). It is found that the identified moving force of loading by the LNN is in good agreement with the actual ones. The large fluctuations exhibited in the forces identified by solving the motion equation of bridges are not observed, especially when the forces move to the intermediate support of the continuous beam. The explicit expressions of the traffic loading are obtained with the LNN rather than by solving the equations of motion. Hence, the ill-posed problem while solving the motion equation inversely does not exist with the proposed method based on the LNN.

However, we still observe significant distinctions between the identified loadings and actual ones at the beginning and the end of the time histories of the loading process (Figure 3a). For the LNN, these distinctions are caused by the impact effects during the loading and unloading processes, which are of extremely short times and thus cannot be accurately described by the Legendre polynomials used in the LNN.

When the speed of loading varies over time (Figure 3b,c), the identified speeds with the LNN are in good agreement with the actual forces in terms of both the peak of the curve and the variation rule. This suggests that the LNN is capable of effectively learning the time-varying features of the moving load. However, significant differences are still observed between the identified and actual speeds at the beginning and the end of the time histories of the loading process.

To evaluate the stability and accuracy of the identified loading using the LNN, the Mean Absolute Error (MAE), Mean Absolute Percentage Error (MAPE), and Root Mean Square Error (RMSE) are calculated for 100 test samples (Table 3). The MAE is the evaluation of Mean Absolute Error between the identified values and true values of the moving load. The MAPE provides the error in terms of the percentage difference between the identified and the applied speed or magnitude of the moving force. The MAPEs are all below 2%, indicating the high accuracy of the load identification using the LNN. Compared to the MAE, the RMSE gives a relatively high weight to large errors by squaring the errors before they are averaged, so the RMSE is sensitive to outliers of the identification results. It is used to reflect the dispersion degree of deviation between the identified and applied moving force. The small RMSE values demonstrate the excellent stability of the LNN. Therefore, the LNN-based methodology proves to be both accurate and stable in identifying the velocity and magnitude of the moving load.

### 4.2. Experimental Verification

The feasibility and accuracy of the LNN in identifying moving loads are validated through experiments with a real continuous beam. To conduct tests of loading with varying parameters, the wheels used to simulate the moving force have two weights of 10 N and 17 N, whose moving speeds are controlled by a stepper motor at 0.5 m/s and 0.375 m/s. At the beginning of the experiment, the wheel moves at a uniform speed under the control of the motor. Upon excitation of the wheel, the beam undergoes vibrations, and its dynamic response are captured by three PZT sensors strategically positioned along the mid-span of the beam’s bottom surface. The voltage time histories from the PZT sensors are recorded using an oscilloscope. Subsequently, the strain distribution within the beam is derived through electromechanical analysis.

**Figure 4:**
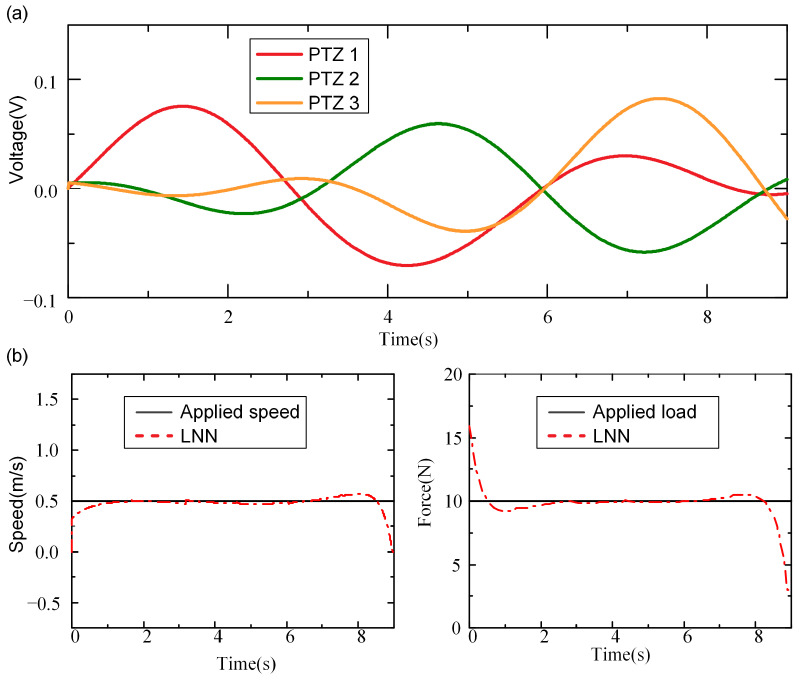
Experimental result of moving force identification using LNN methods: (**a**) the voltages of the PZT sensor arrays; (**b**) the identified moving force.

A loading is assumed to move at a speed of 0.5 m/s with a magnitude of 10 N. The voltages of the PZT sensors are measured, and then the strains of the beam are obtained according to electromechanical analyses (Figure 4a). With the measured dynamic strains, the moving forces are identified with the LNN (Figure 4b). The results indicate that the identified moving force closely matches the applied load, except at the beginning and end of the time histories during the loading process. This discrepancy may be attributed to the impact effects between the wheels and the beam at those specific points in the experiment. Overall, the results successfully demonstrate the feasibility of the LNN approach for identifying moving loads on a real beam bridge.

## 5. Discussions

**Figure 5:**
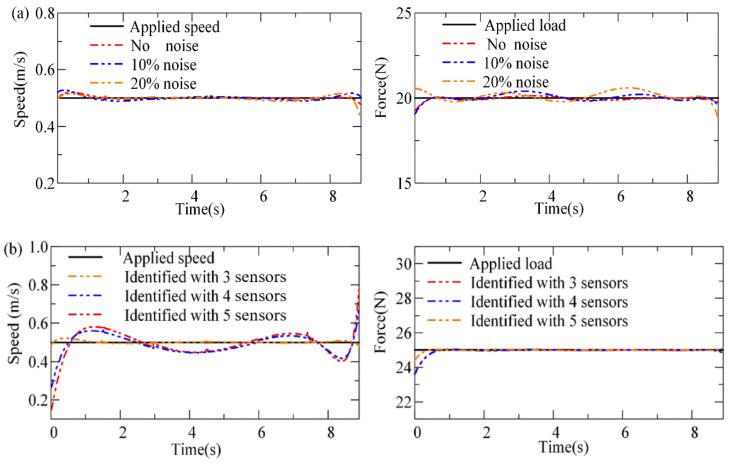
The speed and force of loading with the LNN: (**a**) identified loading with different noise ratios; (**b**) identified speed and force of loading with different numbers of sensors.

To study the influence of noise and sensor quantities on the accuracy of force identification with the LNN, the performances of the LNN for loading identification from sensing signals with different noise levels and sensor quantities are evaluated. The dynamic responses of bridges measured from actual working environments always include noise. Here, two levels of noises are generated using a random process. Then, the strains of the beam with noise added are obtained as follows:

(12) $$\varepsilon =\varepsilon_{\mathrm{simulation}}+E_{P}\cdot N_{\mathrm{noise}\;}\cdot \sigma \left(\varepsilon_{\mathrm{simulation}}\right)$$

where ε denotes the strains of the beam with white noise added, $E_{P}$ denotes the noise ratio, $N_{\mathrm{noise}}$ denotes a vector satisfying the standard normal distribution, and $\varepsilon_{\mathrm{simulation}}$ denotes the strains of the beam. Figure 5a shows the moving load identified with the LNN with different noise levels. It shows that the identified speed by the LNN in all cases accords well with the applied speed, while the deviation of the magnitude of loading grows with the noise level. While the noise level is 20%, the relative errors of the identified magnitude of moving force are less than 10%. Therefore, the method is insensitive to noise interference.

On the other hand, since the data fed into the LNN are the dynamic strains of the beam, the sensor quantities determine the information used for loading identification. Generally, the more information available, the more the network can learn, leading to stronger performance after training. However, when information exceeds a certain threshold, it may lead to overfitting issues, causing the network to excessively fit the training samples. This can result in minimal errors of identified loading with training samples but increased errors with test samples. Herein, the accuracy of the method for load identification in different sensor layout cases is evaluated. The results show that identified forces of loading with the LNN agree well with the applied ones, while the moving speed cannot be identified accurately when the number of sensors increases from three to six (Figure 5b). In conclusion, more sensing signals may not enhance the performance of the LNN.

**Figure 6:**
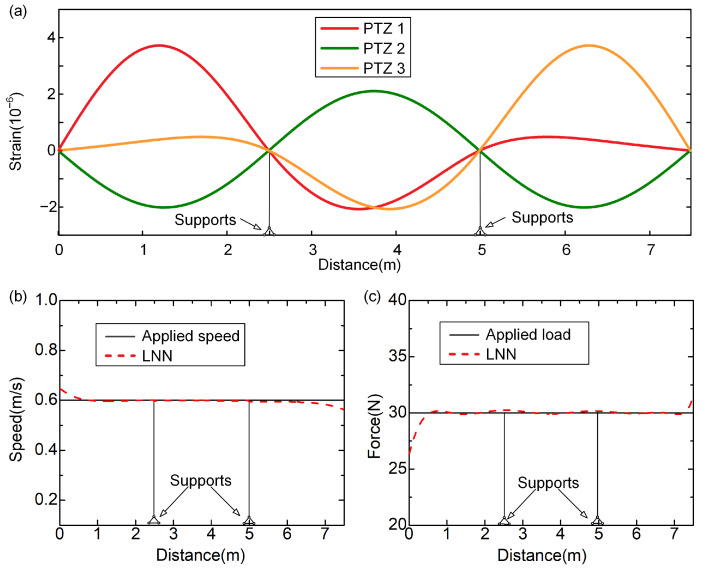
Identified results with the LNN applied in a beam with different lengths: (**a**) the strain responses of the beam; (**b**) identified moving speed of 0.6 m/s; (**c**) identified magnitude of force with 30 N.

To validate the feasibility of the LNN for identifying loads on different bridges of the same type, the well-trained LNN is applied to a different continuous beam. The beam has three spans, each 2.5 m in length, with physical parameters identical to the beam analyzed in Section 3 (Table 1). The loading is assumed to move at a speed of 0.6 m/s with a magnitude of 30 N. The strains of the beam are obtained with the modal superposition method (Figure 6a). When these strains are input into the LNN, the moving forces are identified (Figure 6b,c). It is found that the identified loading with the LNN agrees well with the applied ones. This indicates that the influences of strain response are well understood by the LNN during the training process. Therefore, the explicit expression obtained by the LNN could be applied to different bridges of the same type, which proves the wide applicability of the method.

To further verify the feasibility of the proposed method with the LNN for the identification of multiple moving forces, numerical simulations are conducted with the optimized LNN. In the identification of multiple moving loading, the expression of strain with speed $v_{l}(t)$ and magnitude $p_{l}(t)$ of the multiple moving force to be identified can be calculated as follows:

(13) $$\varepsilon (x,t)=-\sum_{n=1}^{\infty }h\phi_{n}^{\prime \prime }(x)\frac{1}{\mu_{n}\omega_{n}}\int_{0}^{t}\sum_{l=1}^{N_{p}}p_{l}(t)\phi_{n}(v_{l}t)\sin\left[\omega_{n}(t-\tau )\right]d\tau$$

where $p_{l}(t)$ denotes the unknown magnitude of multiple forces and $v_{l}(t)$ is the unknown speed, $l=1,2,\ldots ,N_{p}$.

By inputting Equation (13) into the LNN, we can realize the LNN training process with the database. The database consists of 800 samples, where the parameter values of each group of moving forces are randomly selected from the ranges detailed in Table 2. The dynamic strains of the continuous beam excited by the moving forces are calculated with Equation (13). These dynamic strains serve as the input samples, while the multiple forces constitute the identified samples, forming a sample pair for training the LNN.

**Figure 7:**
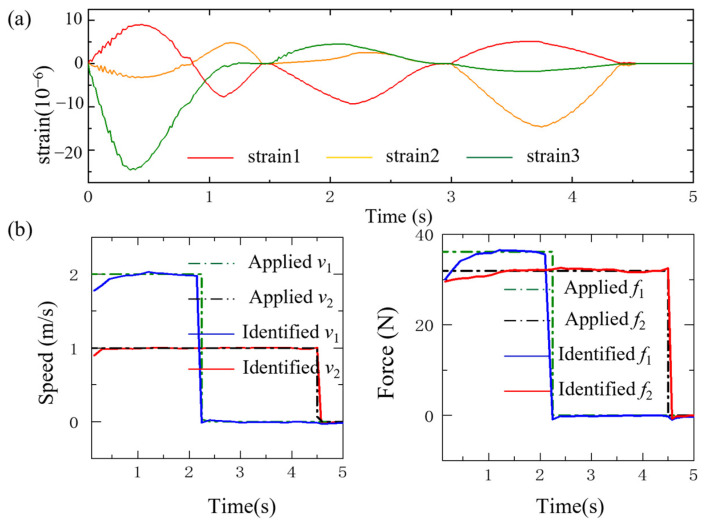
Identified multiple speed and force of loading with the LNN: (**a**) the strains of the beam; (**b**) the identified moving load.

Once the LNN has been effectively trained using the database, the moving forces could be obtained with the strain responses of the beam fed into the trained network. The beam strain response and the comparison of the loads identified by the LNN with the applied loads are illustrated in Figure 7. One loading is assumed to move at a speed of 2 m/s with a magnitude of 36.12 N, and another loading is assumed to move at a speed of 1 m/s with a magnitude of 31.87 N. It is found that both identified moving forces of loading by the LNN are in good agreement with the actual ones. The large fluctuations exhibited in the forces identified by solving the motion equation of bridges are also not observed, especially when the forces move to the intermediate support of the continuous beam. The explicit expressions of the traffic loading are obtained with the LNN rather than by solving the equations of motion. In conclusion, the LNN could be utilized to identify multiple moving forces.

## 6. Conclusions

We propose a novel method utilizing the LNN for moving load identification, enabling the simultaneous identification of both the magnitude and speed of the moving force for various bridges of the same type. Accordingly, the following conclusions could be obtained:

(1) An LNN model is proposed to establish the relationship between bridge strains and traffic load. With the well-trained LNN model, the explicit expression of the moving load could be obtained based on Legendre polynomial combinations. The explicit expressions of the loading may help to reveal the influence of strain response on the moving load and are applicable in a variety of bridges with the same type.
(2) To improve the performance of the LNN, a series of training and optimization of the learning rates and training sample quantities was conducted to determine the optimal values of these hyperparameters. Due to the optimization of hyperparameters of the LNN model, the MPAE of the validation samples with the LNN model achieved lower than 2%, and the training time of the LNN was less than 0.5 h. The outcomes prove the high training efficiency and precision of the method in identification ability.
(3) With the well-trained LNN, the precision of the proposed method was validated through simulations and experiments with a continuous beam. Both the magnitude and moving speed of the loading could be accurately identified at the same time.
(4) The processes of loading identification with the LNN proved to be insensitive to noise and rely on the placement scheme of sensors. When the method was applied in the identification of loading of beam bridges with different lengths and the same other physical parameters, the precisions of identified loading with the LNN were all very high, which confirms its wide applicability.

This paper assumes an idealized scenario in which a concentrated moving load acts on a linear beam. However, in practice, bridges typically experience high traffic flows with numerous vehicles, leading to more complex loading conditions. These conditions exhibit strong time-varying characteristics and significant spatial randomness in vehicle distribution. To identify parameters of multiple vehicles, future research could explore the integration of machine vision algorithms with the proposed model, enabling more precise identification of vehicle parameters such as lateral position, speed, and magnitude. This would better capture the time-varying characteristic of loads in real-world loading scenarios involving multiple vehicles. Additionally, the loads considered in this study are much lower than the real traffic loads encountered on bridges. This discrepancy introduces potential scale effects that may impact the accuracy of the proposed load identification model. To mitigate this, future work could simulate realistic traffic load magnitudes and conduct on-site load testing on actual bridges to validate the accuracy of the model.

## Figures and Tables

**Table 1:** The physical parameters of the continuous beam.

Basic Parameters	Value
Length (*l* = 3 × *l_s_*)	4.5 m = 1.5 m × 3
Young’s modulus (*E_s_*)	200 Gpa
Cross-section (*b_s_* × *h_s_*)	50 mm × 26 mm

**Table 2:** The dataset for verification of the proposed methodology.

	Simulation Dataset	Experimental Dataset
Speed (*v*)	[0, 2] m/s	0.375, 0.5 m/s
Magnitude (*F*)	[10, 50] N	10, 17 N
Sample quantities	800 group	24 group

**Table 3:** Performance of the LNN in the identification of moving force.

Method	MAE		MAPE		RMSE	
	Speed (m/s)	Magnitude (N)	Speed	Magnitude	Speed (m/s)	Magnitude (N)
LNN	0.002	0.078	1.43%	1.52%	0.003	0.207

## Data Availability

The original contributions presented in the study are included in the article; further inquiries can be directed to the corresponding author.
